# Effects of Adipocyte Aryl Hydrocarbon Receptor Deficiency on PCB-Induced Disruption of Glucose Homeostasis in Lean and Obese Mice

**DOI:** 10.1289/ehp.1408594

**Published:** 2015-03-03

**Authors:** Nicki A. Baker, Robin Shoemaker, Victoria English, Nika Larian, Manjula Sunkara, Andrew J. Morris, Mary Walker, Frederique Yiannikouris, Lisa A. Cassis

**Affiliations:** 1 Department of Pharmacology and Nutritional Sciences, and; 2 Division of Cardiovascular Medicine, University of Kentucky, Lexington, Kentucky, USA; 3 Department of Pharmaceutical Sciences, University of New Mexico, Albuquerque, New Mexico, USA

## Abstract

**Background:**

Coplanar polychlorinated biphenyls (PCBs) promote adipocyte inflammation and impair glucose homeostasis in lean mice. The diabetes-promoting effects of lipophilic PCBs have been observed only during weight loss in obese mice. The molecular mechanisms linking PCB exposures to impaired glucose metabolism are unclear.

**Objectives:**

In this study we tested the hypothesis that coplanar PCBs act at adipocyte aryl hydrocarbon receptors (AhRs) to promote adipose inflammation and impair glucose homeostasis in lean mice and in obese mice during weight loss.

**Methods and Results:**

PCB-77 administration impaired glucose and insulin tolerance in LF (low fat diet)–fed control (*AhR^fl/fl^*) mice but not in adipocyte AhR–deficient mice (*AhR^AdQ^*). Unexpectedly, *AhR^AdQ^* mice exhibited increased fat mass when fed a standard LF or high fat (HF) diet. In mice fed a HF diet, both genotypes became obese, but *AhR^AdQ^* mice administered vehicle (VEH) exhibited increased body weight, adipose mass, adipose inflammation, and impaired glucose tolerance compared with *AhR^fl/fl^* controls. Impairment of glucose homeostasis in response to PCB-77 was not observed in obese mice of either genotype. However, upon weight loss, *AhR^fl/fl^* mice administered PCB-77 exhibited increased abundance of adipose tumor necrosis factor-α (TNF-α) mRNA and impaired glucose homeostasis compared with those administered VEH. In contrast, PCB-77 had no effect on TNF-α or glucose homeostasis in *AhR^AdQ^* mice exhibiting weight loss.

**Conclusions:**

Our results demonstrate that adipocyte AhR mediates PCB-induced adipose inflammation and impairment of glucose homeostasis in mice. Moreover, deficiency of AhR in adipocytes augmented the development of obesity, indicating that endogenous ligand(s) for AhR regulate adipose homeostasis.

**Citation:**

Baker NA, Shoemaker R, English V, Larian N, Sunkara M, Morris AJ, Walker M, Yiannikouris F, Cassis LA. 2015. Effects of adipocyte aryl hydrocarbon receptor deficiency on PCB-induced disruption of glucose homeostasis in lean and obese mice. Environ Health Perspect 123:944–950; http://dx.doi.org/10.1289/ehp.1408594

## Introduction

The aryl hydrocarbon receptor (AhR) has established roles in toxicology and phase I drug metabolism (Tijet et al. 2006). Moreover, because mice with whole-body AhR deficiency exhibit organ abnormalities (Abbott et al. 1999; Fernandez-Salguero et al. 1997; Harstad et al. 2006; Lahvis et al. 2005; Vasquez et al. 2003), endogenous ligand activation of this receptor has been implicated in the control of proliferation and/or differentiation of various cell types. Several studies have demonstrated marked sequestration of xenobiotic ligands of AhR, including lipophilic coplanar polychlorinated biphenyls (PCBs) (Brown and Lawton 1984; Fukano and Doguchi 1977; McFarland and Clarke 1989), in adipose tissue. Rather than serve as an inert storage reservoir for PCBs (Bourez et al. 2012, 2013), adipocyte AhR activation by coplanar PCB AhR ligands promoted adipose inflammation (Arsenescu et al. 2008; Kim et al. 2012). In addition, administration of coplanar PCBs to lean mice impaired glucose and insulin tolerance, and these effects were abolished by an AhR antagonist (Baker et al. 2013b). Impaired glucose homeostasis in mice exposed to coplanar PCBs was associated with an adipose-specific increase in expression of tumor necrosis factor-α (TNF-α), a cytokine linked to impairment of insulin-stimulated glucose uptake. These results suggest that in addition to serving as a storage reservoir, adipocytes respond to PCBs to promote inflammation and negatively influence glucose homeostasis.

Due to bioaccumulation in adipose lipids, the total body burden of PCBs is increased in obese rodents and humans (Kim et al. 2011; Myre and Imbeault 2014; Pelletier et al. 2003). Interestingly, in contrast to lean mice, in which administration of PCB-77 impaired glucose and insulin tolerance (Baker et al. 2013b), mice that were obese from consumption of a high-fat (HF) diet showed no effect of PCB-77 on glucose or insulin tolerance. In contrast, during periods of weight loss, PCB concentrations in adipose tissue decrease and serum concentrations of PCBs increase (Chevrier et al. 2000; Irigaray et al. 2006). Moreover, when obese mice administered PCB-77 were subjected to weight loss, they exhibited impairments in glucose and insulin tolerance that blunted the beneficial effects of weight loss (Baker et al. 2013b). One possible explanation for this observation is that hydrophobic PCBs are released upon lipolysis within adipocytes during weight loss to act systemically, or to alternately interact with the cytosolic AhR within adipocytes.

In this study, we hypothesized that coplanar PCBs promote insulin resistance and impair glucose homeostasis through adipocyte-specific AhR activation. To test this hypothesis, we generated mice with adipocyte AhR deficiency (*AhR^AdQ^*). Because coplanar PCBs impair glucose homeostasis in lean mice, we defined the role of adipocyte AhR on PCB-induced impairment of glucose and insulin tolerance in mice fed a low-fat (LF) diet. Moreover, because the bioaccumulation of lipophilic PCBs is altered with obesity and upon weight loss, we examined the role of adipocyte AhR during the development of HF diet–induced obesity and during weight loss in obese mice previously exposed to PCB-77.

## Materials and Methods

*Chemicals*. 3,3´,4,4´-Tetrachlorobiphenyl (PCB-77) was purchased from AccuStandard Inc. (New Haven, CT).

*Animal treatments and sample collection*. All experiments met the approval of the Animal Care and Use Committee of the University of Kentucky. Animals used in this study were treated humanely and with regard for the alleviation of suffering. Mice were maintained in individually ventilated cages (Sealsafe Plus Mouse IVC Green Line; Tecniplast) with an *ad libitum* automated water system (Endstrom), with aspen wood chip bedding (Harlan Teklad Sani-Chips; Harlan Laboratories) and cotton nesting squares (Neslet; Ancare) containing a small amount of shredded paper (Enviro-Dri; Shepherd Specialty Papers) for enrichment. Conditions included a light/dark cycle of 14 hr/10 hr, a temperature of 70°C (± 2°C), and humidity ranging from 30 to 70%. AhR-floxed (*AhR^fl/fl^*) mice with loxP sites flanking exon 2 were a generous gift of M. Walker (University of New Mexico) (Agbor et al. 2011). Female *AhR^fl/fl^* mice were bred to hemizygous transgenic male Cre mice under control of an adiponectin/promoter/enhancer [B6;FVB-Tg(Adipoq-cre)1Evdr/J; The Jackson Laboratory]. Male *AhR^fl/fl^* littermate controls and *AhR^AdQ^* mice were used in all experiments. There were no overt differences in health or appearance between genotypes at the start of the study. Mice of each genotype were randomly assigned to study groups for each specific experiment. A total of 86 mice were used in these experiments, of which *n* = 37 were *AhR^fl/fl^*, and *n* = 49 were *AhR^AdQ^*. Of the total 86 mice, *n* = 27 were administered vehicle (VEH; tocopherol-stripped safflower oil), and *n* = 28 mice were administered PCB-77.

In experiments confirming the efficiency and specificity of adipocyte AhR deletion, male mice of each genotype (*n* = 5/genotype) were fed standard mouse diet (Harlan Teklad 2918 Global Rodent Diet, irradiatedU; Harlan Laboratories) *ad libitum* from weaning to 2 months of age.

In experiments examining lean mice, male mice (2 months of age) of each genotype were administered VEH or PCB-77 [50 mg/kg; by oral gavage at approximately 1000 hours given once in week 1 and once in week 2; *n* = 3–8 mice/group (Baker et al. 2013b)] and fed an LF diet (10% kcal as fat, D12450B; Research Diets) *ad libitum*. Mice in each group were examined 48 hr after the last administered dose (week 2).

For experiments on obese mice, mice (2 months of age) were fed an HF diet (60% kcal as fat, D12492; Research Diets) *ad libitum* for 12 weeks to promote the development of obesity. Mice of each genotype were administered VEH or PCB-77 (50 mg/kg; by oral gavage at approximately 1000 hours given as four doses in weeks 1, 2, 9, and 10; *n* = 6–8 mice/group). For weight loss experiments, mice of each genotype and treatment group were placed on the HF diet for 12 weeks and then changed to the LF diet for 4 weeks to induce weight loss.

For all experiments, body weights were quantified weekly at approximately 0900 hours. At the study end point (approximately 0600 hours), mice were transferred to the investigator’s laboratory using a stainless steel cart, and then anesthetized [ketamine/xylazine, 10/100 mg/kg, by intraperitoneal (i.p.) injection beginning at approximately 1100 hours] for exsanguination and tissue harvest [liver, subcutaneous adipose (SubQ), retroperitoneal adipose (RPF), epididymal adipose (EF), interscapular brown adipose tissue (BAT), kidney, brain, and heart].

*Measurement of body composition*. Body composition of mice was determined by nuclear magnetic resonance spectroscopy [EchoMRI (magnetic resonance imaging)] at baseline (before diet administration) and at study end point. Briefly, conscious mice were placed in clear, cylindrical plastic tubes (sized by animal weight) at approximately 1000 hours. The tubes have holes for breathing and are maintained in a horizontal plane during the procedure. The scanner applies an external magnetic field at a level of 5-gauss beyond the surface of the system in a 9-in. radius from the edge of the animal holder. An equal field is present on the opposite side of the system. Three sequential scans (taking approximately 2 min each) were conducted for each animal. Following completion of the scan, the tubes were cleaned with soap and water using a long bottle brush. For this measurement, mice were transported using a stainless steel cart to a procedure room within the Division of Laboratory Animal Resources (DLAR) and then returned to the room where they were housed using the same cart.

*Glucose (GTT) and insulin (ITT) tolerance tests*. Mice were fasted for 4 or 6 hr for ITT or GTT, respectively, and blood glucose concentrations were measured using the tail vein with a handheld glucometer (Freedom Freestyle Lite; Abbott Laboratories). For the GTT, mice were transferred to a procedure room within the DLAR and injected i.p. with d-glucose (Sigma; 20% in saline, 10 μL/g of body weight at approximately 1300 hours); blood glucose concentrations were quantified at 0, 15, 30, 60, 90, and 120 min. For ITT, mice were transferred to a procedure room within DLAR, injected i.p. with human insulin [Novolin (Nordisk), 0.0125 μM in saline/g of body weight at approximately 1400 hours], and blood glucose concentrations were quantified at 0, 30, 60, 90, and 120 min. Total area under the curve (AUC; arbitrary units) was calculated as previously described (Baker et al. 2013b).

*Quantification of PCB-77 and hydroxylated metabolites in tissues*. Tissue samples (EF, liver) were weighed and homogenized in distilled H_2_O (dH_2_O). Cold acetonitrile and 50 μL of internal standard (10 μM ^13^C-labeled d6-PCB-77) were added to homogenates, which were vortexed, sonicated, and centrifuged at 15,000 rpm. Supernatants were transferred to glass vials, and pellets were reextracted with cold acetonitrile, with the process repeated twice. Pooled supernatants were dried under nitrogen and reconstituted in 100 μL of 99/1 methanol/dH_2_O containing 0.5% formic acid and 0.1% ammonium formate. PCB-77 and hydroxy-PCB-77 were measured using liquid chromatography-tandem mass spectrometry as described previously (Baker et al. 2013a).

*Extraction of RNA and quantification of mRNA abundance using real-time polymerase chain reaction (PCR)*. Total RNA was extracted from tissues (liver, adipose tissue, soleus muscle) using the SV Total RNA Isolation System kit (Promega Corporation) according to the manufacturer’s instructions. RNA concentrations were determined using a NanoDrop 2000 spectophotometer (Thermo Scientific). cDNA was synthesized from 0.4 μg total RNA with qScript cDNA SuperMix (Quanta Biosciences) in the following reaction: 25°C for 5 min, 42°C for 30 min, and 85°C for 5 min. The cDNA was diluted to 0.4 ng/μL and amplified with an iCycler (Bio-Rad) and Perfecta SYBR Green Fastmix for iQ (Quanta Biosciences). Using the difference from *GAPDH* rRNA (reference gene) and the comparative Ct method, the relative quantification of gene expression in each sample was calculated. Primers (Eurofins MWG Operon) were designed using Primer-BLAST (http://www.ncbi.nlm.nih.gov/tools/primer-blast/). The PCR reaction was as follows: 94°C for 5 min, 40 cycles at 94°C for 15 sec, 58°C or 64°C (based on tested primer efficiency) for 40 sec, 72°C for 10 min, and 100 cycles from 95°C to 45.5°C for 10 sec. Primer sequences were as follows: *AhR*, forward 5´-GACC​AAAC​ACAA​GCTA​GACT​TCAC​ACC, reverse 5´-CAAG​AAGC​CGGA​AAAC​TGTC​ATGC; *Cyp1A1*, forward 5´-AGTC​AATC​TGAG​CAAT​GAGT​TTGG​-3´, reverse 5´-GGCAT​CCAG​GGAA​GAGT​TAGG-3´; *GAPDH*, forward 5´-GCCA​AAAG​GGTC​ATCA​TCTC-3´, reverse 5´-GGCC​ATCC​ACAG​TCTT​CT-3´; *Tnf-alpha*, forward 5´-CCCA​CTCT​GACC​CCTT​TACT​C-3´, reverse 5´-TCAC​TGTC​CCAG​CATC​TTGT-3; F4/80, forward 5´-CTTT​GGCT​ATGG​GCTT​CCAG​TC-3´, reverse 5´-​GCAA​GGAG​GACA​GAGT​TTAT​CGTG-3´.

*Quantification of adipocyte size and cell number*. Sections of formalin-fixed (10% wt/vol) pieces of SubQ tissue were stained with hematoxylin and eosin. Images of slides were taken at 10× magnification. Using the “detect edges” setting, image threshold, and object count features of NIS Elements software (Nikon Instruments Inc.), we quantified the area of each adipocyte and the number of adipocytes within a 700 × 700 μm measurement frame. Adipocyte size was calculated on three measurement frames within each section of adipose tissue per mouse from three mice per group.

*Differentiation of adipocytes from the stromal vascular fraction (SVF) of SubQ tissue.* SubQ tissue was taken from the inguinal region, minced, and incubated in basal medium (Zenbio) supplemented with collagenase (1 mg/mL) and penicillin/streptomyocin (5%) for 1 hr with shaking at 37°C, as previously described (Rodbell 1964). Two days after SVF cells had reached 100% confluence, media was changed to differentiation medium (OM-DM; Zenbio) and changed every other day for 8 days. Cells were harvested for RNA using TRIzol (Life Technologies); cDNA synthesis and real-time PCR were performed as described above. The SVF was harvested at 100% confluence prior to addition of the adipocyte differentiation medium, and again at day 8 after differentiation (Adipo fraction).

*Statistical analysis*. Data are presented as mean ± SE. Data were log transformed prior to statistical analysis. A two-way analysis of variance (ANOVA; SigmaPlot, version 12.0; Systat Software Inc.) was used to determine statistical significance, which was defined as *p* < 0.05. GTT and ITT results were analyzed using repeated measure two-way ANOVA. The Holm-Sidak method was used for post hoc analyses.

## Results

**Figure 1:**
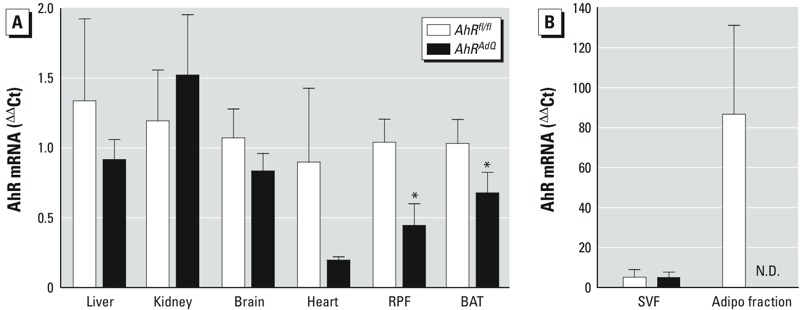
Comparison of AhR-deficient mice (*AhR^AdQ^*) with *AhR^fl/fl^* controls. (*A*) *AhR* mRNA abundance in liver, kidney, brain, heart, retroperitoneal fat (RPF), and brown adipose tissue (BAT); data are mean ± SE from *n *= 5 mice/genotype. *p < 0.05 compared with *AhR^fl/fl^*. (*B*) *AhR* mRNA abundance in SVF (preadipocytes) and after 8 days of differentiation to mature adipocytes (adipo fraction) in cells from *AhR^fl/fl^* and *AhR^AdQ^* mice fed standard mouse diet; data are mean ± SE from *n *= 3 mice/genotype. N.D., not detected.

*Generation and characterization of mice with adipocyte AhR deficiency*. To confirm effective and specific deletion of exon 2 of AhR in adipocytes, we quantified *AhR* mRNA abundance in adipose tissues (SubQ, RPF, EF, BAT), liver, brain, heart, and kidney from mice fed standard laboratory diet (2 months of age). *AhR* mRNA abundances were not significantly different in liver, kidney, or brain from *AhR^fl/fl^* compared with *AhR^AdQ^* mice (Figure 1A). In the heart, *AhR* mRNA abundance was reduced in *AhR^AdQ^* compared with *AhR^fl/fl^* mice but did not meet criteria for statistical significance (Figure 1A; *p* = 0.17). In RPF white adipose tissue and BAT of *AhR^AdQ^* mice, *AhR* mRNA abundance was significantly decreased compared with *AhR^fl/fl^* mice (Figure 1A; *p* < 0.05). Because adipose tissue is heterogeneous in cell types, preadipocytes were differentiated from the SVF of SubQ tissue of mice from each genotype. *AhR* mRNA abundance was not significantly altered in preadipocytes from *AhR^AdQ^* mice prior to differentiation (SVF, Figure 1B). In contrast, *AhR* mRNA abundance was significantly reduced in fully differentiated adipocytes from *AhR^AdQ^* mice compared with *AhR^fl/fl^* controls (Adipo fraction, Figure 1B; *p* < 0.05).

Unexpectedly, 2-month-old male *AhR^AdQ^* mice fed standard mouse diet exhibited significantly increased fat mass and reduced lean mass compared with age-matched *AhR^fl/fl^* controls (Table 1). In addition, *AhR^AdQ^* mice had significantly larger visceral adipose depots (EF and RPF; *p* < 0.05) and moderately larger SubQ depots (*p* = 0.45; Table 1). However, these effects did not result in significant differences in body weights between genotypes (Table 1). Because differences in body fat deposition could potentially affect glucose homeostasis, we conducted baseline GTTs and ITTs. There was no significant difference in glucose or insulin tolerance between genotypes in *AhR^AdQ^* mice compared with *AhR^fl/fl^* controls (see Supplemental Material, Figure S1A,B).

**Table 1:** Characteristics of *AhR^fl/fl^* and *AhR^AdQ^*mice fed standard diet.

Parameter/genotype	*AhR*^*fl/fl*^	*AhR*^*AdQ*^
Body weight (g)	24.6 ± 0.4	23.6 ± 0.5
Lean mass (% body weight)	81.0 ± 0.5	77.2 ± 1.2*
Fat mass (% body weight)	8.9 ± 0.3	11.2 ± 0.9*
Epididymal fat (EF) (g)	0.89 ± 0.12	1.27 ± 0.07*
Retroperitoneal fat (RPF) (g)	0.16 ± 0.02	0.26 ± 0.03*
Subcutaneous fat (SubQ) (g)	0.56 ± 0.08	0.74 ± 0.03
Data are mean ± SE from* n *= 6–8 mice/group. *p < 0.05 compared with *AhR*^*fl/fl*^.		

**Figure 2:**
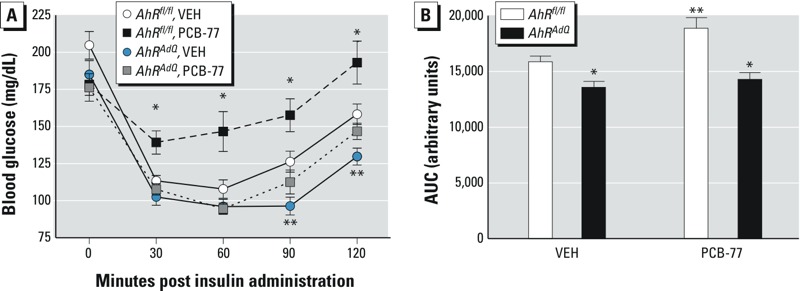
Effect of adipocyte AhR deficiency on insulin tolerance in lean mice administered PCB-77. (*A*) Blood glucose concentrations following administration of insulin in mice of each genotype administered VEH or PCB-77 (week 3). Data are mean ± SE from *n *= 6–8 mice/group. *p < 0.05 compared with VEH within genotype. **p < 0.05 compared with *AhR^fl/fl^* within treatment. (*B*) Area under the curve (AUC) for data in (*A*). Data are mean ± SE from *n *= 6–8 mice/group. *p < 0.05 compared with *AhR^fl/fl^* within treatment. **p < 0.05 compared with VEH within genotype.

*Effect of adipocyte AhR deficiency on insulin tolerance in lean mice acutely exposed to PCB-77*. We previously demonstrated that lean mice exposed to four divided doses of PCB-77 develop impaired insulin tolerance within 48 hr after the last dose (Baker et al. 2013b). Thus, we defined effects of adipocyte AhR deficiency on acute PCB-77–induced dysregulation of insulin tolerance in mice fed an LF diet (Figure 2A,B). Body weights were not significantly different between groups at the study end point (*AhR^fl/fl^* VEH, 28.1 ± 0.5; *AhR^fl/fl^* PCB-77, 27.9 ± 0.5; *AhR^AdQ^* VEH, 25.5 ± 0.5; *AhR^AdQ^* PCB-77, 27.1 ± 0.5 g; *p* > 0.05). Compared with *AhR^fl/fl^* VEH mice, *AhR^AdQ^* VEH mice showed a modest, but significant, improvement in insulin tolerance, as indicated by a significant reduction in blood glucose concentrations at 90 and 120 min after insulin administration and by a significant reduction in the AUC (Figure 2A,B; *p* < 0.05). Notably, administration of PCB-77 significantly impaired insulin tolerance in *AhR^fl/fl^* but not in *AhR^AdQ^* mice (Figure 2A,B; *p* < 0.05).

**Figure 3:**
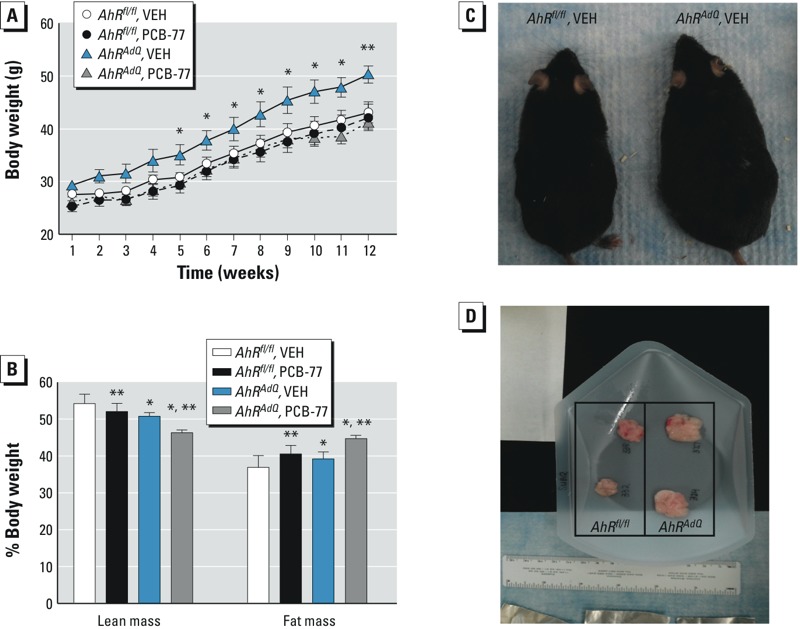
Effect of adipocyte AhR deficiency on the development of obesity and increased adiposity in mice fed the HF diet for 12 weeks. (*A*) Body weights of mice by treatment group and genotype during the 12 weeks of HF feeding; data are mean ± SE from *n *= 6–8 mice/treatment/genotype. *p < 0.05 compared with *AhR^fl/fl^* within treatment. (*B*) Lean mass and fat mass as a percentage of body weight; data are mean ± SE from *n *= 6–8 mice/group. *p < 0.05 compared with *AhR^fl/fl^* within treatment group. **p < 0.05 compared with VEH within genotype. (*C*) A representative mouse from each genotype. (*D*) Subcutaneous adipose tissue removed from representative *AhR^fl/fl^* (left) and *AhR^AdQ^* (right) mice (*n *= 2/group).

**Figure 4:**
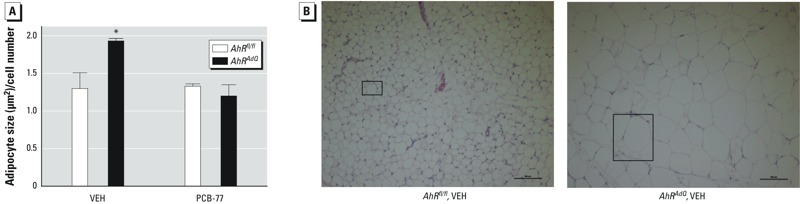
Effect of adipocyte AhR deficiency on the size of subcutaneous adipocytes of mice fed the HF diet. (*A*) Quantification of adipocyte size in tissue sections of subcutaneous adipose tissue. Adipocyte size was calculated from three measurement frames within each section of adipose tissue (*n *= 3 sections/mouse) from *n *= 3 mice/group. *p < 0.05 compared with *AhR^fl/fl^* within treatment. (*B*) Representative sections of subcutaneous adipose tissue from mice of each genotype administered VEH; boxes denote a single adipocyte within each representative section.

*Effect of adipocyte AhR deficiency on the development of obesity, body fat distribution, and glucose homeostasis*. Because obesity has been reported to increase the total body burden of lipophilic PCBs (Brown and Lawton 1984; Fukano and Doguchi 1977; McFarland and Clarke 1989), we examined effects of adipocyte AhR deficiency on the development of HF diet–induced obesity and glucose homeostasis in mice of each genotype administered VEH or PCB-77. When challenged with an HF diet, adipocyte AhR–deficient *AhR^AdQ^* mice administered VEH had significantly increased body weights, reduced lean mass, and increased fat mass compared with *AhR^fl/fl^* controls (Figure 3A–C; *p* < 0.05). Moreover, excess adiposity in HF-fed *AhR^AdQ^* mice administered VEH was deposited subcutaneously, with significant increases in SubQ tissue mass (*AhR^fl/fl^*, 3.8 ± 0.5; *AhR^AdQ^*, 5.5 ± 0.4; *p* < 0.05; representative images in Figure 3D) and adipocyte size compared with *AhR^fl/fl^* controls (Figure 4A,B). However, these effects were not observed in *AhR^AdQ^* mice administered PCB-77 (Figure 4A). Moreover, in mice administered VEH, mRNA abundance of F4/80, a macrophage marker, was significantly increased in EF tissue of *AhR^AdQ^* mice compared with *AhR^fl/fl^* controls, regardless of treatment group (VEH groups: *AhR^fl/fl^*, 1.62 ± 0.67; *AhR^AdQ^*, 3.52 ± 0.68; *p* < 0.05; PCB-77 groups: *AhR^fl/fl^*, 1.29 ± 0.49; *AhR^AdQ^*, 2.12 ± 0.68 ^ΔΔ^Ct; *p* < 0.05). In liver or soleus muscle from HF-fed mice administered VEH, there was no significant effect of genotype on *AhR* mRNA abundance (see Supplemental Material, Figure S2; *p* = 0.06). However, administration of PCB-77 resulted in a significant increase in *AhR* mRNA abundance in liver but not in soleus muscle from HF-fed mice of each genotype, with no significant differences between genotypes (see Supplemental Material, Figure S2; *p* < 0.05).

Consistent with previous findings (Baker et al. 2013b), PCB-77 had no significant effect on glucose tolerance in obese *AhR^fl/fl^* or *AhR^AdQ^* mice (see Supplemental Material, Figure S3A). At 12 weeks of HF feeding in PCB-77–treated mice of each genotype, adipose tissue concentrations of PCB-77 were considerably higher than those of its metabolite hydroxy-PCB-77 (weight gain phase; see Supplemental Material, Figure S3B,C). Moreover, adipose concentrations of PCB-77 were significantly higher in *AhR^AdQ^* mice compared with *AhR^fl/fl^* mice. In contrast, adipose concentrations of hydroxy-PCB-77 were not significantly different between genotypes (see Supplemental Material, Figure S3C).

*Effect of adipocyte AhR deficiency on PCB-77–induced impairments of glucose and insulin tolerance in obese mice exhibiting weight loss*. A previous study (Baker et al. 2013b) demonstrated that when obese mice lost weight, benefits of weight loss to improve glucose and insulin tolerance were diminished in mice exposed to PCB-77 during the weight-gain phase of diet-induced obesity. Thus, obese *AhR^fl/fl^* and *AhR^AdQ^* mice administered VEH or PCB-77 during the weight-gain phase (12 weeks of HF feeding) were made to lose weight by switching to an LF diet (4 weeks). Despite higher body weights of VEH-treated HF-fed *AhR^AdQ^* mice compared with VEH-treated *AhR^fl/fl^* controls prior to weight loss (Figure 3), after 4 weeks of the LF diet, there were no significant differences in body weights between the groups (see Supplemental Material, Figure S4).

**Figure 5:**
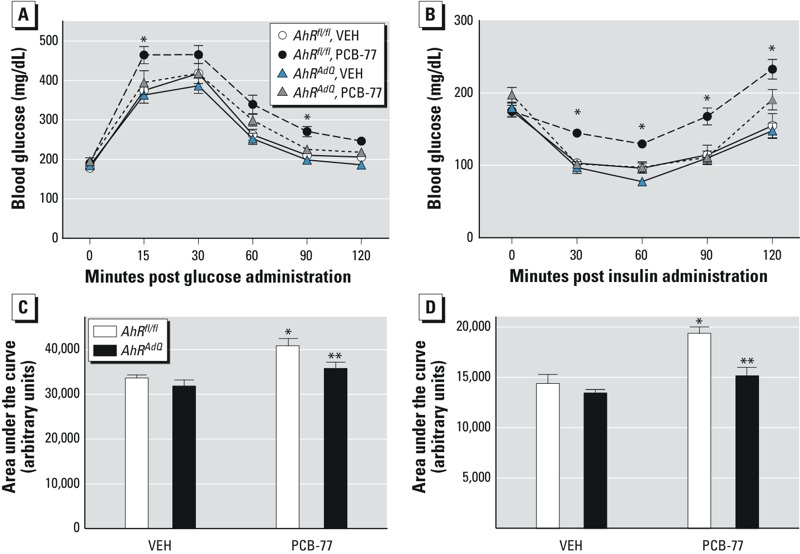
Effect of adipocyte AhR deficiency on PCB-77-induced impairment of glucose and insulin tolerance in obese mice exhibiting weight loss. After 12 weeks of HF feeding, mice of each genotype and treatment group were switched to an LF diet for 4 weeks to induce weight loss. (*A*) Blood glucose concentrations following a bolus of i.p.-administered glucose in mice of each genotype and treatment group following 4 weeks of weight loss. (*B*) Blood glucose concentrations following a bolus of i.p.-administered insulin in mice of each genotype and treatment group. For (*A*) and (*B*), *p < 0.05 compared with *AhR^fl/fl^* within treatment group. (*C*) Area under the curve (AUC) for data in (*A*) and (*D*) AUC for data in (*B*); data are mean ± SE from *n *= 6–8 mice/genotype/treatment group. *p < 0.05 compared with VEH within genotype. **p < 0.05 compared with *AhR^fl/fl^* within treatment group.

As we demonstrated previously (Baker et al. 2013b), after weight loss *AhR^fl/fl^* PCB-77 mice exhibited significantly impaired glucose and insulin tolerance compared with *AhR^fl/fl^* VEH mice (Figure 5A–D; *p* < 0.05). In contrast, PCB-77 had no effect on glucose or insulin tolerance in *AhR^AdQ^* mice experiencing weight loss. In response to weight loss, concentrations of PCB-77 in adipose tissue decreased significantly in both genotypes (see Supplemental Material, Figure S3B; *p* < 0.05). Although concentrations of hydroxy-PCB-77 increased in adipose tissue of both genotypes of mice after experiencing weight loss, levels of the PCB metabolite were significantly decreased in adipose tissue from *AhR^AdQ^* mice compared with *AhR^fl/fl^* controls (see Supplemental Material, Figure S3C; *p* < 0.05).

**Figure 6:**
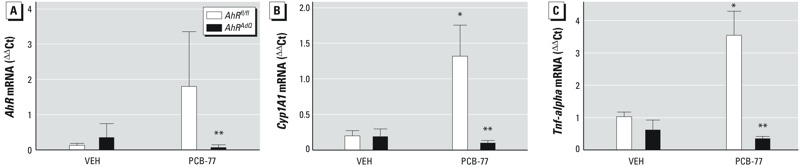
Effect of adipocyte AhR deficiency and PCB-77 on mRNA abundance of *AhR* (*A*), *Cyp1A1* (*B*), and *Tnf‑alpha* (*C*) in epididymal adipose tissue from mice of each genotype and treatment group following 4 weeks of weight loss. Data are mean ± SE from *n *= 5 mice/group. *p < 0.05 compared with VEH within genotype. **p < 0.05 compared with *AhR^fl/fl^* within treatment group.

Previous studies demonstrated that administration of PCB-77 during the weight-gain phase of diet-induced obesity had no effect on adipose inflammation or glucose homeostasis, presumably due to sequestration of the lipophilic toxicant in expanded adipose lipids (Baker et al. 2013b). However, when obese mice previously exposed to PCB-77 lost weight, the benefits of weight loss to reduce adipose mRNA abundance of TNF-α and to improve glucose tolerance were mitigated (Baker et al. 2013b). These results suggest that upon weight loss, PCB-77 is released from expanded adipose lipids of obese mice to promote AhR-mediated adipose inflammation and to impair glucose tolerance, blunting beneficial effects of weight loss. We quantified mRNA abundance of AhR, CYP1A1 (as a marker of AhR activation) and TNF-α (as a marker of inflammation) in adipose tissue of obese mice experiencing weight loss (mice were exposed to PCB-77 during the weight-gain phase of diet-induced obesity). In mice administered PCB-77, *AhR* mRNA abundance in adipose tissue was significantly decreased in *AhR^AdQ^* mice compared with *AhR^fl/fl^* mice (Figure 6A; *p* < 0.05). Similarly, in adipose tissue of *AhR^fl/fl^* mice administered PCB-77, *Cyp1A1* mRNA abundance was markedly increased, indicative of AhR activation (Figure 6B; *p* < 0.05). In contrast, PCB-77 had no effect on *Cyp1A1* mRNA abundance in adipose tissue from mice with adipocyte AhR deficiency. Finally, *Tnf*-*alpha* mRNA abundance was significantly increased in adipose tissue of *AhR^fl/fl^* mice administered PCB-77 compared with VEH, with no effect of the toxicant on *Tnf*-*alpha* mRNA abundance in adipose tissue of *AhR^AdQ^* mice (Figure 6C; *p* < 0.05).

## Discussion

Results from these experiments demonstrate a pivotal role for adipocyte AhRs in the regulation of adiposity and regional adipose deposition, and in the effects of lipophilic coplanar PCBs to impair glucose homeostasis in mice. Because of their lipophilicity, PCBs bioaccumulate in adipose lipids. Our results support the concept that adipose tissue is not simply an inert reservoir of these toxicants. Rather, bioaccumulation of PCBs within adipocyte lipids, with dynamic redistribution of PCBs upon lipolysis (e.g., with weight loss), results in activation of cytoplasmic AhR to impair glucose utilization and/or insulin signaling. Indeed, adipocyte AhR deficiency totally abolished acute effects of coplanar PCBs to impair insulin tolerance in lean mice, and also abolished effects of PCBs to impair glucose homeostasis in obese mice experiencing weight loss. Moreover, we observed an unexpected phenotype of increased body weight and adiposity in HF-fed mice that lack AhR expression in adipocytes (*AhR^AdQ^*). Interestingly, with increased adiposity, *AhR^AdQ^* mice exhibited significant SubQ tissue deposition. Despite SubQ tissue deposition, overall increases in adiposity resulted in pronounced adipose inflammation and more significant impairments of glucose homeostasis in HF-fed *AhR^AdQ^* mice. Because these effects were observed in HF-fed adipocyte AhR mice that were not exposed to environmental toxicants, these results suggest that an endogenous ligand(s) of AhR plays a role in the regulation of regional adiposity, adipose inflammation, and glucose homeostasis. Moreover, these results demonstrate that adipocyte AhRs mediate effects of coplanar PCBs to impair glucose homeostasis in lean mice and in obese mice experiencing weight loss.

Previous studies in our laboratory demonstrated that coplanar PCB ligands of the AhR promoted inflammation of mature murine 3T3-L1 adipocytes (Arsenescu et al. 2008; Baker et al. 2013a, 2013b). Similarly, exogenous AhR ligands such as dioxin, as well as coplanar PCBs, promoted expression of a variety of inflammatory genes in human adipocytes (Kim et al. 2012). Moreover, administration of dioxin to C57BL/6 mice increased adipose tissue inflammation (Kim et al. 2012). Results from the present study demonstrate that deficiency of adipocyte AhR promotes adipose inflammation, including infiltration of macrophages into adipose tissue (as detected by F4/80 expression). Moreover, these results extend previous findings by demonstrating that a coplanar PCB acts at adipocyte AhR to increase expression of the proinflammatory cytokine TNF-α in adipose tissue as well as to impair insulin resistance in C57BL/6 mice. Notably, acute effects of PCB-77 to impair glucose homeostasis in lean mice, and in obese mice experiencing weight loss, were abolished in mice with adipocyte AhR deficiency. These results demonstrate that PCB-induced alterations in glucose homeostasis are mediated through adipocyte AhR. Selective effects of lipophilic toxicants such as PCBs at adipocyte AhR may relate to their marked bioaccumulation within adipose lipids (Bourez et al. 2012, 2013).

An unexpected finding of the present study was an increase in body weight, adiposity, and impaired glucose homeostasis in *AhR^AdQ^* mice fed an HF diet (i.e., the VEH group). Anorexia and/or body wasting have been described as toxic manifestations of dioxin exposure (Tuomisto et al. 1995). In addition, dioxin has been demonstrated to reduce differentiation of 3T3-L1 adipocytes in an AhR-dependent manner (Alexander et al. 1998). However, effects of AhR activation appear to be concentration dependent, as previous studies in our laboratory demonstrated that low concentrations (< 0.1 nM) of dioxin, as well as coplanar PCBs (3.4 μM), promote differentiation of 3T3-L1 adipocytes, whereas higher concentrations of these AhR ligands (10 nM dioxin and 34 μM coplanar PCBs) inhibited adipocyte differentiation (Arsenescu et al. 2008). In addition to regulation of adipocyte differentiation, mouse embryonic fibroblasts (either as preadipocytes or differentiated to adipocytes) from mice with whole-body AhR deficiency exhibited elevated triglyceride synthesis (Alexander et al. 1998). Results from the present study demonstrate that, in the setting of diet-induced obesity, deficiency of adipocyte AhR results in increased adipocyte size and adiposity. It is unclear if adipocyte AhR deficiency stimulated differentiation of new adipocytes (Arsenescu et al. 2008) or enhanced triglyceride synthesis to promote adiposity. Moreover, AhR-mediated regulation of body weight may result from the combined effects of activation of AhR in various cell types because congenic mice with high AhR signaling activity were more susceptible to diet-induced obesity compared with those with low AhR activity (Kerley-Hamilton et al. 2012). Further studies are warranted to define cell-specific mechanisms for AhR regulation of adipose mass and body weight.

In addition to an increase in overall adiposity and body weight when challenged with an HF diet, adipocyte AhR–deficient male mice (*AhR^AdQ^*) exhibited enhanced deposition of SubQ tissue. Overall increases in adiposity of obese adipocyte AhR–deficient mice resulted in more pronounced impairments of glucose homeostasis. Estrogen has been associated with a gynoid lower body distribution of adipose tissue (subcutaneously) in females, whereas androgen may contribute to more upper body (visceral) adipose deposition in males. The AhR interacts with estrogen and its receptors through various mechanisms, including potential estrogenic effects in the absence of estrogen, versus antiestrogenic effects in the presence of estrogen (Brunnberg et al. 2011). It is possible that alterations in adipose deposition in HF-fed adipocyte AhR–deficient mice may result from either an estrogenic or antiandrogenic effect to promote gynoid adipose deposition. Further studies are required to define the basis of changes in regional adiposity in adipocyte AhR–deficient mice fed an HF diet.

Coplanar PCBs are substrates for *Cyp1A1*, a target gene of AhR activation, resulting in the production of water-soluble hydroxylated metabolites that are more readily excreted (Toborek et al. 1995). Results from the present study demonstrate that during the weight gain phase of diet-induced obesity, PCB-77 is not readily metabolized to hydroxy metabolites in adipocytes. This is most likely the result of sequestration of the parent toxicant to adipocyte triacylglycerol droplets, where CYP1A1 does not have ready access to the substrate. Similar to previous reports (Kim et al. 2011), levels of PCB-77 in adipose tissue were increased in adipocyte AhR–deficient mice exhibiting increased body weight and adiposity. It is possible that elevations in adipose levels of the parent toxicant, PCB-77, contributed to an ability of the toxicant to act at other target sites important in the control of body weight, adipose mass, and the regulation of glucose homeostasis. For example, in the present study, adipocyte AhR–deficient mice exposed to PCB-77 did not exhibit increased adiposity or body weight, suggesting that increased levels of the parent toxicant acted at AhR on other cell types to protect against obesity and impair glucose homeostasis. In support, exposure of C57BL/6 mice fed a very high-fat diet to persistent organic pollutants that included AhR ligands resulted in paradoxical improvements in insulin and glucose tolerance (Ibrahim et al. 2011). As previously indicated (Ibrahim et al. 2011) the interplay between dietary nutrients and environmental pollutants is complex; however, our results suggest that actions of PCBs at adipocyte AhR mediate harmful effects of the toxicant to impair glucose homeostasis.

In the present study, upon weight loss in *AhR^fl/fl^* mice, levels of the hydroxy-PCB-77 metabolite in adipose tissue increased markedly and were associated with a marked induction of adipose CYP1A1. In contrast, adipose tissue from *AhR^AdQ^* mice experiencing weight loss did not exhibit increased abundance of CYP1A1, which most likely contributed to reduced adipose levels of the hydroxy-PCB-77 metabolite. These results demonstrate that CYP1A1 induction by PCB-77 in adipose tissue, mediated by adipocyte AhR, are associated with increased levels of hydroxy-PCB-77.

## Conclusions

Results from this study demonstrate that coplanar PCBs act at adipocyte AhR to promote adipose inflammation and impair glucose homeostasis in lean mice and in obese mice experiencing weight loss. Moreover, results demonstrate a previously unappreciated role for adipocyte AhR to regulate adiposity, adipose inflammation, body weight, and glucose homeostasis in mice with diet-induced obesity. These results suggest that bioaccumulation of lipophilic coplanar PCBs to adipose tissue contributes to the development of adipocyte insulin resistance. Moreover, endogenous AhR ligands, present in the setting of diet-induced obesity, may play a pivotal role in regulating adipose mass and deposition.

## Supplemental Material

(374 KB) PDF
