# Supplementary material for: Effects of Adipocyte Aryl Hydrocarbon Receptor Deficiency on PCB-Induced Disruption of Glucose Homeostasis in Lean and Obese Mice
Source: Environ Health Perspect. 2015 Mar 3;123(10):944–50. doi: 10.1289/ehp.1408594 (PMC4590748; doi:10.1289/ehp.1408594)
Supplement: (374 KB) PDF [file ehp.1408594.s001.acco.pdf]

Environ Health Perspect
doi: 10.1289/ehp.1408594

Note to Readers: *EHP* strives to ensure that all journal content is accessible to all readers. However, some figures and Supplemental Material published in *EHP* articles may not conform to 508 standards due to the complexity of the information being presented. If you need assistance accessing journal content, please contact ehp508@niehs.nih.gov. Our staff will work with you to assess and meet your accessibility needs within 3 working days.

Supplemental Material

Effects of Adipocyte Aryl Hydrocarbon Receptor Deficiency on PCB-Induced Disruption of Glucose Homeostasis in Lean and Obese Mice

Nicki A. Baker, Robin Shoemaker, Victoria English, Nika Larian, Manjula Sunkara, Andrew J. Morris, Mary Walker, Frederique Yiannikouris, and Lisa A. Cassis

Figure S1. Deficiency of AhR in adipocytes has no effect on glucose (A) or insulin (B) tolerance in mice fed standard mouse diet. *AhR*$^{fl/fl}$ and *AhR*^{AdQ} mice fed standard diet were administered a bolus of glucose (A) or insulin (B) and then blood glucose concentrations were quantified at various times. Data are mean ± SEM from n = 8-10/mice/genotype.

Figure S2. AhR mRNA abundance in liver and soleus muscle from HF-fed (12 week) *AhR*$^{fl/fl}$ and *AhR*^{AdQ} mice administered vehicle (VEH) or PCB-77. Data are mean ± SEM from n = 6-8 mice/group. *, P<0.05 compared to VEH within genotype.

Figure S3. Adipocyte AhR deficiency impairs glucose tolerance in HF-fed mice administered vehicle (VEH). Concentrations of PCB and its hydroxy metabolite in adipose tissue of obese mice before (12 weeks of HF feeding, weight gain) and after weight loss. **(**A), Blood glucose concentrations were quantified following a bolus of glucose administered intraperitoneally to mice of each genotype and treatment group at 12 weeks of HF feeding. Data are mean ± from n = 6-7 mice/group/treatment. *, P<0.05 compared to *AhR*$^{fl/fl}$ within treatment. Adipose tissue concentrations of PCB-77 (B) and its hydroxy metabolite (C) before and after weight loss in mice of each genotype and treatment group. Data are mean ± SEM from n = 6-8 mice/group. *, P<0.05 compared to *AhR*$^{fl/fl}$ within time point. **, P < 0.05 compared to 12 weeks within genotype.

Figure S4. Body weights at study endpoint in *AhR*$^{fl/fl}$ and *AhR*^{AdQ} mice administered VEH or PCB-77 during the weight gain phase of HF feeding (12 weeks), followed by switching to a LF diet to induce weight loss for 4 weeks. Data are mean ± SEM from n = 6 mice/genotype/treatment group.

A

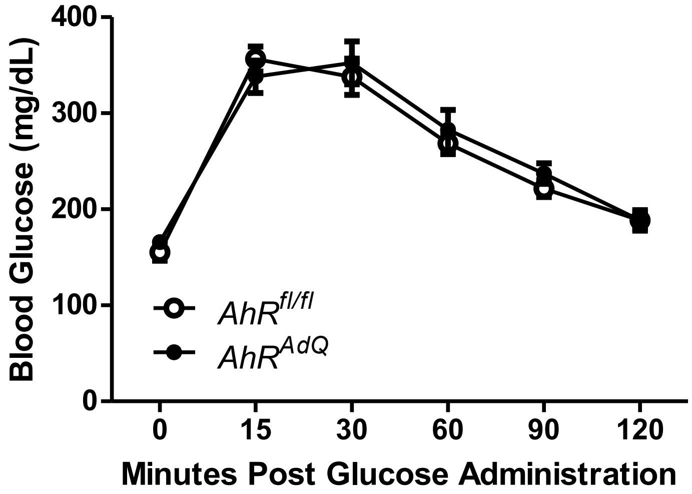

B

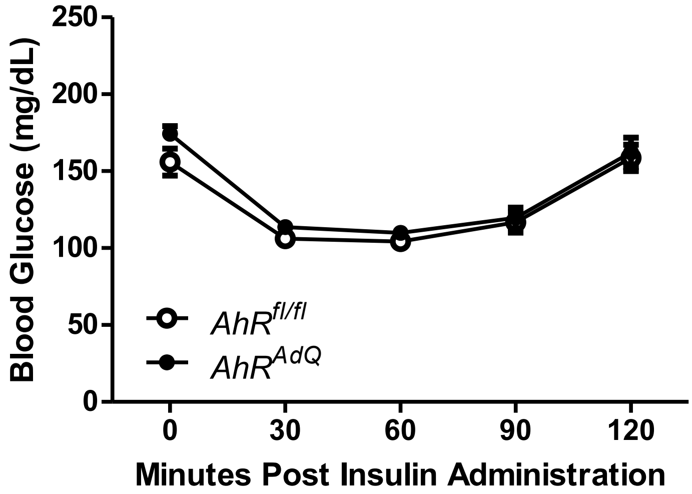

Figure S1. Deficiency of AhR in adipocytes has no effect on glucose (A) or insulin (B) tolerance in mice fed standard mouse diet. *AhR*$^{fl/fl}$ and *AhR*^{AdQ} mice fed standard diet were administered a bolus of glucose (A) or insulin (B) and then blood glucose concentrations were quantified at various times. Data are mean ± SEM from n = 8-10/mice/genotype.

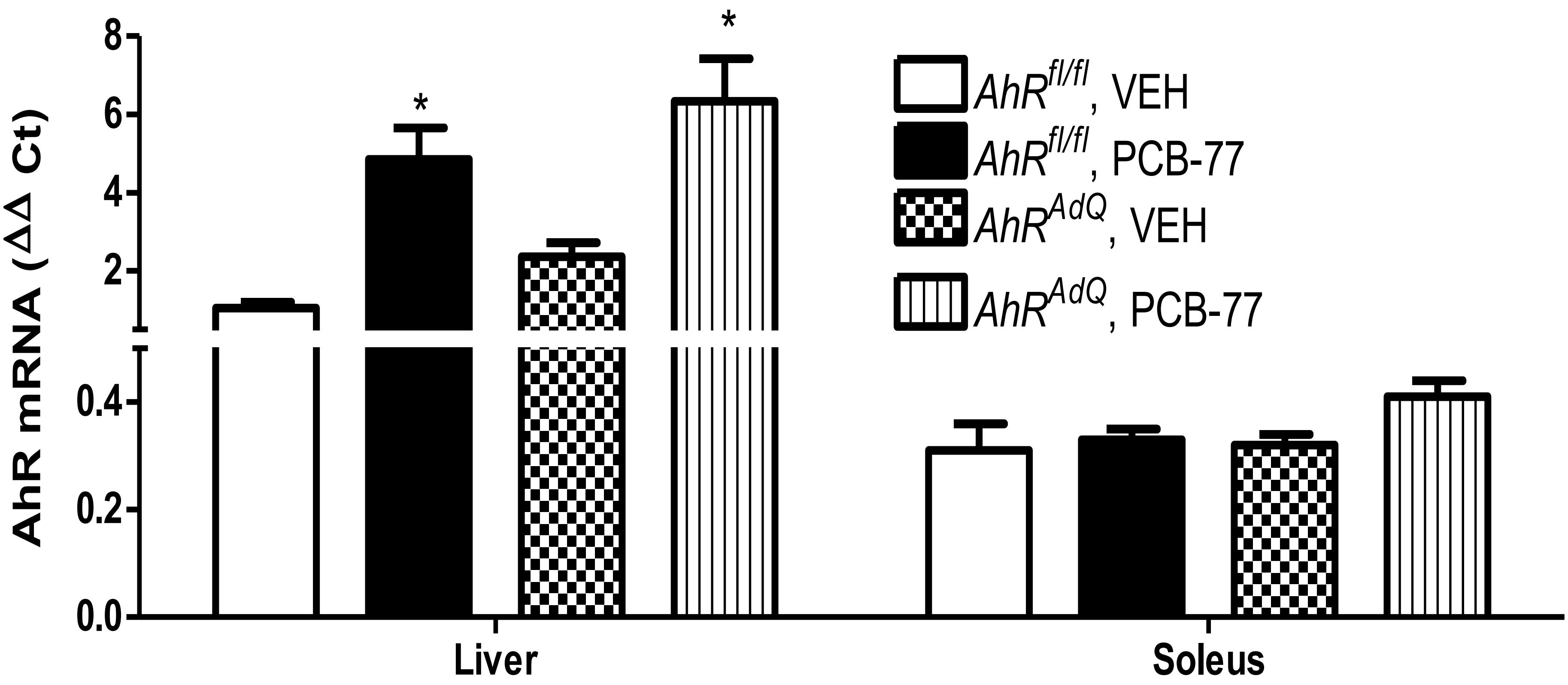

Figure S2. AhR mRNA abundance in liver and soleus muscle from HF-fed (12 week) $AhR^{fl/fl}$ and AhR^{AdQ} mice administered vehicle (VEH) or PCB-77. Data are mean ± SEM from n = 6-8 mice/group. *, P<0.05 compared to VEH within genotype.

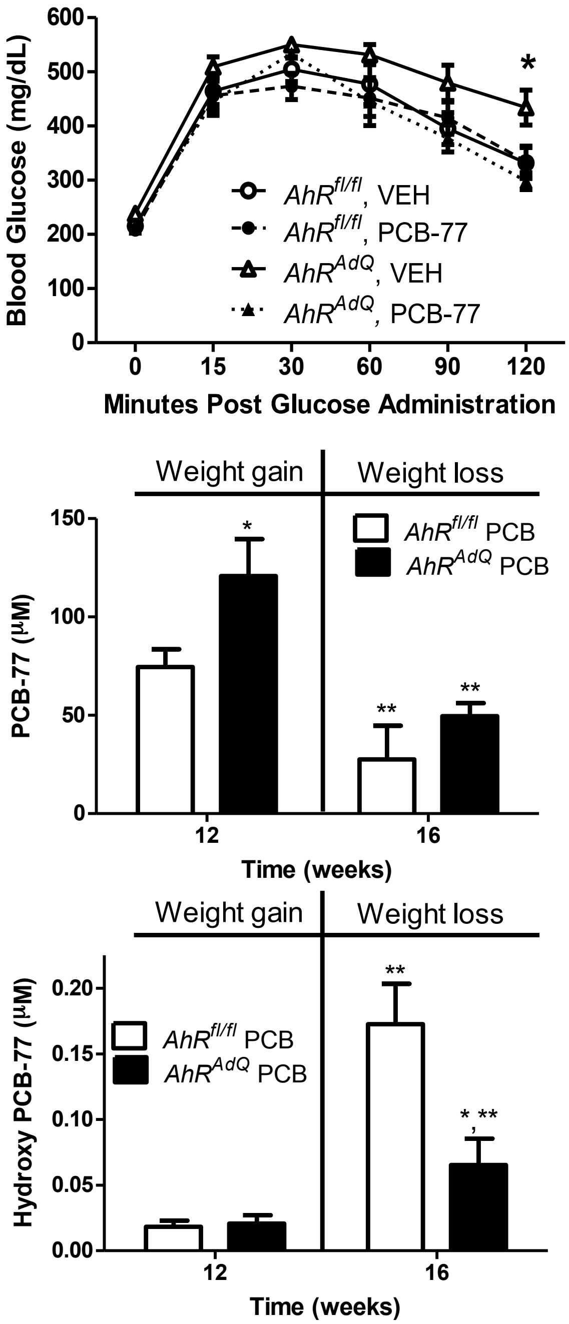

Figure S3. Adipocyte AhR deficiency impairs glucose tolerance in HF-fed mice administered vehicle (VEH). Concentrations of PCB and its hydroxy metabolite in adipose tissue of obese mice before (12 weeks of HF feeding, weight gain) and after weight loss. (A), Blood glucose concentrations were quantified following a bolus of glucose administered intraperitoneally to mice of each genotype and treatment group at 12 weeks of HF feeding. Data are mean ± from n = 6-7 mice/group/treatment. *, P<0.05 compared to $AhR^{fl/fl}$ within treatment. Adipose tissue concentrations of PCB-77 (B) and its hydroxy metabolite (C) before and after weight loss in mice of each genotype and treatment group. Data are mean ± SEM from n = 6-8 mice/group. *, P<0.05 compared to $AhR^{fl/fl}$ within time point. **, P < 0.05 compared to 12 weeks within genotype.

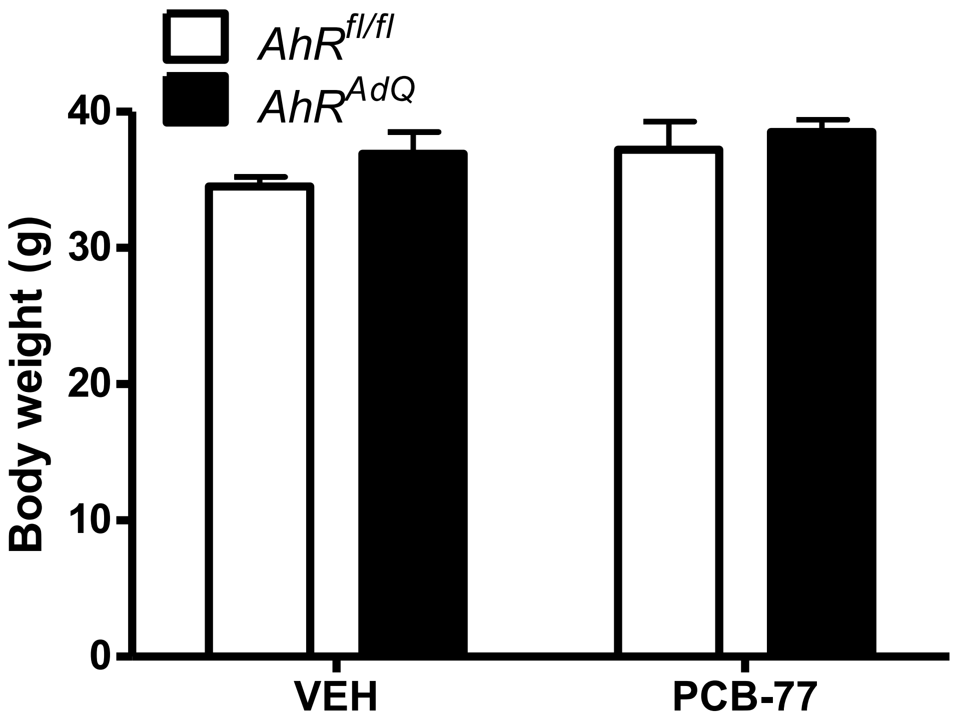

Figure S4. Body weights at study endpoint in *AhR*$^{fl/fl}$ and *AhR*^{AdQ} mice administered VEH or PCB-77 during the weight gain phase of HF feeding (12 weeks), followed by switching to a LF diet to induce weight loss for 4 weeks. Data are mean ± SEM from n = 6 mice/genotype/ treatment group.
